# Motor modules in robot-aided walking

**DOI:** 10.1186/1743-0003-9-76

**Published:** 2012-10-08

**Authors:** Leonardo Gizzi, Jørgen Feldbæk Nielsen, Francesco Felici, Juan C Moreno, José L Pons, Dario Farina

**Affiliations:** 1 Pain Clinic, Center for Anesthesiology, Emergency and Intensive Care Medicine, University Hospital Göttingen, Göttingen, Germany; 2 Department of Neurorehabilitation Engineering, Bernstein Center for Computational Neuroscience, University Medical Center Göttingen, Georg-August University, Von-Siebold-Str, 4,37075, Göttingen, Germany; 3 Department Human Movement and Sport Sciences, University of Roma Foro Italico, Piazza Lauro De Bosis 6, Rome, 00196, Italy; 4 Regionshospitalet Hammel Neurocenter, Aarhus University, Voldbyvej 15, 8450, Hammel, Denmark; 5 Bioengineering Group, Spanish National Research Council, CSIC, Carretera Campo Real, Madrid, Spain

**Keywords:** Motor modules, Robotic gait trainer, Motor control

## Abstract

**Background:**

It is hypothesized that locomotion is achieved by means of rhythm generating networks (central pattern generators) and muscle activation generating networks. This modular organization can be partly identified from the analysis of the muscular activity by means of factorization algorithms. The activity of rhythm generating networks is described by activation signals whilst the muscle intervention generating network is represented by motor modules (muscle synergies). In this study, we extend the analysis of modular organization of walking to the case of robot-aided locomotion, at varying speed and body weight support level.

**Methods:**

Non Negative Matrix Factorization was applied on surface electromyographic signals of 8 lower limb muscles of healthy subjects walking in gait robotic trainer at different walking velocities (1 to 3km/h) and levels of body weight support (0 to 30%).

**Results:**

The muscular activity of volunteers could be described by low dimensionality (4 modules), as for overground walking. Moreover, the activation signals during robot-aided walking were bursts of activation timed at specific phases of the gait cycle, underlying an impulsive controller, as also observed in overground walking. This modular organization was consistent across the investigated speeds, body weight support level, and subjects.

**Conclusions:**

These results indicate that walking in a Lokomat robotic trainer is achieved by similar motor modules and activation signals as overground walking and thus supports the use of robotic training for re-establishing natural walking patterns.

## Background

The description and understanding of a complex task such as walking have been challenging researchers for centuries [1]. Animal models suggest the major role of dedicated neural circuitries responsible for the rhythmic muscular activity during locomotion determined by a rhythm generating network (central pattern generators, CPG) and a muscle weighting network, the latter being devoted to determine the activation of muscles based on the target and sensory input( [2-6]). The rhythmic activity of locomotion can be described by means of a quantitative representation based on motor modules (also referred to as *muscle synergies* or *muscle weightings*) and activation signals (also called *primitives* or *factors*). The identification of motor modules is obtained by factorization of the muscular activation signals and has been applied for the description of animal natural behavior [7,8], upper body movements in healthy humans [9] and in stroke patients [10], and during human locomotion [11-15]. A series of studies have identified a small number of activation signals that can explain the muscular activation during human locomotion, for both treadmill [14] and overground walking at different velocities [13], different body weight support levels [11], stepping and hopping, walking on a slippery surface [16] and running [17]. The most consistent finding of these studies is the presence of a burst-like activation of motor modules during the gait cycle [18]. This impulsive control of motor modules, which interestingly is preserved also in stroke [19,20] and partly in spinal cord injured patients [21], is consistent with a neuronal network in which the timing of activity generated by central pattern generator neurons is directed to the motoneurons via a premotor network that distributes the activity to motoneurons in a task dependent manner, determined by sensory and descending control information [3].

The recovery of walking is a crucial aspect of rehabilitation, improving the quality of life and patient’s independence. For this reason, robot-aided walking is considered a promising tool for gait rehabilitation [22] in stroke [23,24], multiple sclerosis [25,26], spinal cord and brain injury [27,28], Parkinson’s disease [29,30], and cerebral palsy [31].

The level of engagement of the patients is important for the success of the rehabilitation process [32-34]. For example, Hornby and colleagues [35] assessed that continuous sagittal plane assistance on robotic gait training administered to chronic stroke patients is not as effective as therapist-based training. Together with the conclusion of that study, recent results [36-38] are suggestive of a more effective recovery of function obtained when the patient does not receive passively the movement from the machine but rather actively contributes, so that the robot helps and sustains the task only where and when motor deficiencies are present (“assistance as needed” approach).

However, although a number of studies have described the kinematic output of the robot-patient system and the functional improvement of robot-aided gait rehabilitation [26,30,39-41], there are less data documenting the muscular synergistic activation patterns during walking aided by a robot. This information is of essential importance for the use of robot-aided walking in rehabilitation since it is necessary to prove if similar muscle control strategies are elicited even in mechanically different condition. The limitation to the sagittal plane of the human-machine interaction, the non-completely transparent behavior of the machine -due to its mass and control-response inertia-, the different proprioceptive feedback, together with discomfort due to body weight support (BWS) may interfere with normal motor control even in healthy subjects.

Therefore, the aim of this study was first to assess the presence of a modular organization of walking in healthy subjects during the use of a robot-aided rehabilitation device. The specific focus was on verifying the hypothesis that walking in a robotic rehabilitation device can be described by a small number of motor modules which are controlled in an impulsive, burst-like way, by activation signals, as in overground walking [20]. Second, we aimed to verify that motor modules and activation signals are independent of speed and BWS level, once the trajectories are fixed to a physiological gait pattern [42] and the contribution of the machine to movement is set to the minimal intervention and to assess similarity of motor control with respect of overground walking. To verify these hypotheses, healthy subjects walked at different speeds and BWS levels in a Lokomat (Hocoma, Zurich, Switzerland), which is a driven gait orthosis specifically designed to physically guide repetitive, rhythmic, bilateral lower extremity movements (for an accurate description of the machine see [42,43]).

## Materials and methods

### Subjects

Eight healthy subjects (3 women, 5 men, age 35.8±9.0 yrs, stature 171.2±6.7 cm, weight 67.0±9.0 kg) volunteered in this experiment. All volunteers involved in this study reported no history of neurologic or orthopedic diseases that could interfere with locomotion and had no previous experience of robot-aided walking. Approval for the study was obtained by the local ethics committee.

### Procedures

The subjects were asked to walk overground and in the Lokomat. The sequence of free and robot-aided walking was randomized. For overground trials, the subjects were asked to walk at a self-selected low speed along a 6-m straight line, overground walking for at least 5 times. A low speed was requested to the subjects in order to approximately match the middle of the range of speeds of walking with the robot (see Result section). No further indications were administered to the subjects in order to maintain the overground walking as natural as possible. A minimum of 30 gait cycles per subject was extracted from these tests for further analyses.

For robot-aided locomotion trials, the subjects were interfaced with a Lokomat orthosis. The device was adjusted so that the hip and knee centers lined up with the joint centers of the subject at best, according to subject’s feedback on comfort; a BWS harness was mounted on the subject. All subjects were accustomed by the same experienced physical therapist. The ankle joint was free to move naturally (although the treatment with this robot usually involves an elastic bandage to avoid foot-drop in neurological patients) in order to simulate as closely as possible the free walking condition. Subjects were then asked to walk normally. During a 10-min familiarization session with the robot, fine adjustments to mechanical (cuffs and support position) and kinematic (range of motion) parameters were made in order to match the subject’s natural walking pattern. After familiarization, the subjects were asked to keep walking naturally in the robot at speeds ranging from 1 to 3km/h (0.5km/h increment) and BWS levels of 0, 15% and 30%. Each walking trial lasted at least 1 min. Speed and BWS level were randomized for each trial spanning all the combinations of selected values. Prior to each recording, the subjects familiarized for a few minutes with the new speed/BWS settings. The Lokomat machine guidance force (i.e., the force input required to the subject to initiate the movement) was set to 0% in all tests; this means that the machine was following the movement of the subject without interfering. A value of 0% guidance force is a free run mode in which exoskeleton joints are easily moveable.

At the final stage of treatment, the patients involved in robotic rehabilitation with the Lokomat are usually able to walk in the range of speeds and BWS investigated in this study with a guidance force lower than 30%, with 0% representing the ideal condition of recovery [24].

A low guidance force (simulating that patient apparently walks without an orthosis) could be suitable for patients with hemiparesis needing unilateral guidance only. In such mode the patient has to bear the weight that is not supported directly by the body weight support system. The inertia of the machine is compensated via a combination of cooperative Path Control strategy (in which the subject is allowed to influence the timing of movement along a physiological walking pattern) and automatic treadmill speed adaptation (see [42-44] for details). The subjects were asked to walk comfortably at the set velocity. The last 30 gait cycles for each condition were selected for the analysis.

### EMG

Surface EMG signals were recorded in bipolar derivation with pairs of Ag/AgCl electrodes (Ambu® Neuroline 720 01-K/12, Ambu A/S, Ballerup, Denmark), placed with 22 mm of centre-to-centre spacing. Before electrode placement, the skin was shaved, if needed, and gently abraded with abrasive paste. EMG signals were amplified with gain 2000 (EMG-USB, LISiN – OT Bioelettronica, Torino, Italy), band-pass filtered (8^th^ order Bessel filter, bandwidth 10–750 Hz), sampled at 2048 Hz, and A/D converted on 12 bits. A reference electrode was placed on the subject’s wrist.

A total of 16 muscles (8 per body side) were investigated: Tibialis Anterior (TA), Gastrocnemius Medialis (GM), Soleus (SOL), Vastus Lateralis (VL), Rectus Femoris (RF), Biceps Femoris (BF), Rectus Abdominis (RA), and Erector Spinae (ES). Electrodes for EMG recordings were placed according to the SENIAM recommendations [45] for all muscles, except for RA (not described by SENIAM) that was analyzed with electrodes positioned following the recommendations of Ng et al. [46].

### Kinematics during overground walking

For the overground walking tests, the kinematics of locomotion was acquired by means of a VICON stereophotogrammetry system (Vicon Motus, Vicon Motions Systems, Centennial, CO), capturing frames at 100 samples/s. Four markers were located on each foot at the ankle, toe, and heel (the Plug-in-gait, Vicon Motion Systems Ltd., Oxford, UK), and at the base of the big toe.

Foot kinematics (i.e. detection of minima Z component of heel markers) was used to separate strides during walking trials. A stride was identified as the period between two heel strikes on the same side. The stride starting and ending samples were marked on a timeline; stride duration, cadence and speed were computed using a VICON built-in algorithm for the extraction of stride parameters. Kinematics and EMG recordings were synchronized offline.

### Kinematic and dynamic data during-robot aided walking

For the tests in the Lokomat, the knee angle and force exchanged against the machine at the knee joint were recorded from the analog output box of the Lokomat. Heel contacts for left and right foot were identified by means of the Lokomat integrated infrared system, which provides a square wave signal with a rising front at the heel strike instant (i.e. when the heel of the subject interrupts the infrared line on the sensor). Stride identification was used for signal segmentation in gait cycles.

### Signal analysis

Electromyographic signals were segmented for each gait cycle, as identified from the kinematics data (overground walking) or from the Lokomat output, and band-pass filtered (4^th^ order zero-lag Butterworth digital filter, pass-band 20–400 Hz) to attenuate DC offset, motion artifacts, and high frequency noise [45]. The filtered signals were full-wave rectified and low-pass filtered (4^th^ order, cut off frequency 10 Hz) to obtain the muscular activation patterns. Signals were then time-interpolated to 200 samples per segment. Although the relative amplitude activation of synergistic muscles (GM and SOL, for example) may vary at changing of body weight, BWS or body mass [47], with the aim of enhancing the structural properties of muscular activation the envelope of each muscle signal was normalized by its maximal value for each stride [9,11,12,19,20].

The EMG signal envelopes recorded from *M* muscles are indicated as:

(1) $$X\left(k\right)={\left[x_{1}\left(k\right),x_{2}\left(k\right),\cdots ,x_{M}\left(k\right)\right]}^{T}$$

where *x*_*m*_*(k)* is the activity of the *m*th muscle at the time instant *k*. The activation signals are indicated with *P*(*k*) and are less than the number of muscles (*N<M*):

(2) $$P\left(k\right)={\left[p_{1}\left(k\right),p_{2}\left(k\right),\cdots ,p_{N}\left(k\right)\right]}^{T}$$

The muscle activities are obtained from the activation signals by linear transformation with gain factors *s*_*mn*_. The matrix whose columns are the weights of each activation signal for each muscle is denoted as *S* and referred to as the matrix of motor modules [48]. The relation between *X(k)* and *P(k)* is described as follows:

(3) $$X\left(k\right)\approx X_{r}\left(k\right)=S\cdot P\left(k\right)$$

where *X*_*r*_(*k*) is the muscle activity vector reconstructed by the matrix of motor modules and the activation signals.

To take into account the inter-subject and trial-to-trial variability, the extraction of motor modules was performed concatenating the trials from each subject for each condition [19,20]. Legs were treated separately and only results from the left leg are reported since the results from the two legs were not statistically different. The non-negative matrix factorization (NMF) algorithm was applied to extract the matrix *S* of motor modules and the activation signals *P(k)* Eq. (3) from the normalized data [48-50]. Modules were extracted according to the model in Eq. (3). The number of motor modules needed for accurate description of the movement was assessed by the dimensionality analysis proposed by d’Avella et al. [51]. According to this procedure, the quality in reconstruction of the muscle activation pattern is analyzed as a function of the number of modules and the minimum number of modules is identified as the point in which this curve changes slope (for details, see [51]).The reconstruction quality was assessed by means of the Variation Accounted For (VAF) index defined as VAF = 1 – SSE/SST, where SSE (sum of squared errors) is the unexplained variation and SST (total sum of squares) is the total variation (of the data) [19,20]. Together with the criterion proposed by d’Avella and colleagues [51,52], a minimal VAF value of 80% was also required in this study to consider the reconstruction quality as satisfactory.

The matrices of motor modules extracted from each individual were compared among individuals and conditions by computing the average of scalar product between modules (i.e., pairs of columns of the matrix *S*) and normalizing by the product of the norms of the columns (referred through the text as *mean similarity of motor modules*) [9,51]. Because vectors of modules are non-negative, this operation provides a value that ranges between 0 and 1. The degree of similarity between activation signals was computed as the peak value of the cross-correlation function at zero lag [20]. Before the cross-correlation was computed, the activation signals were ordered to obtain the maximal similarity with the Gaussian-like waveforms proposed by Ivanenko et al. [11]. Motor modules were ordered following the association with the respective activation signals. In order to compare the angle and force profiles among speeds and BWS levels, kinematic and dynamic data were segmented and time-interpolated to 200 samples, according to the procedure performed on sEMG signals. Angular and force values for knee joint are reported in the Results section.

### Statistical analysis

Once verified the non-normality of the data distribution (Shapiro-Wilk test), non-parametric analysis was performed to assess differences in similarity of motor modules and correlation of activation signals with respect to overground walking in different conditions of robotic aided walking. The Friedman test with Schaich and Hamerle post-hoc correction when necessary, was performed in Matlab. Significance level was set to 0.05.

## Results

**Figure 1:**
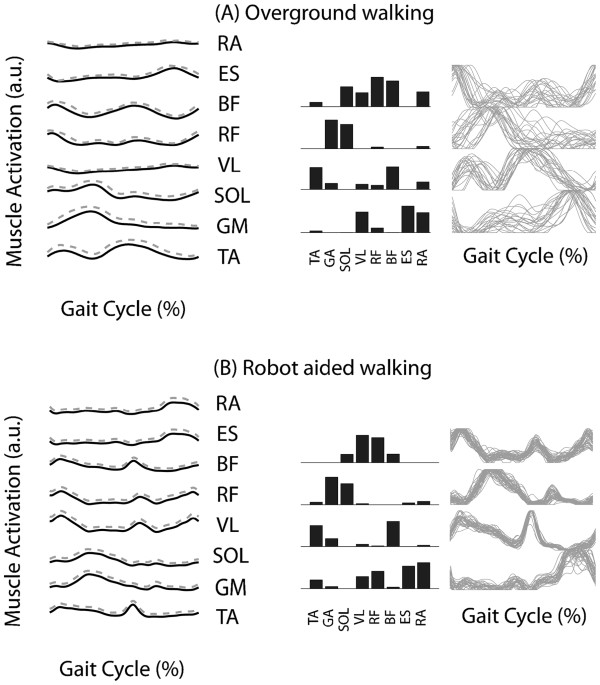
**Data from a representative subject. **Rectified, low pass filtered and averaged surface EMG signals (left) solid line - mean, dashed line - standard deviation), motor modules and activation signals for overground walking (right) (**A**) and robot-aided walking (**B**) at comparable speed (2.0km/h) and body weight support (0%). Motor modules and activation signals are extracted from the concatenation of gait cycles; each grey line represents the contribution of each cycle. The analysis evidences that the impulsive structure of motor control typical of locomotion is maintained during robot-aided walking, as reflected by the activation signals (gray lines on the right). Motor modules during robot-aided walking (black histograms in the center of the figure) resulted similar to those extracted during overground walking.

All the subjects walked comfortably in the robot spanning the ranges of velocities and BWS levels. None of the subjects reported discomfort or pain during walking in the robot rehabilitation machine. Figure 1 shows the factorization process to extract motor modules during locomotion for a representative subject for both overground walking and robot-aided walking at 2.0km/h 0% BWS.

### Overground walking

**Figure 2:**
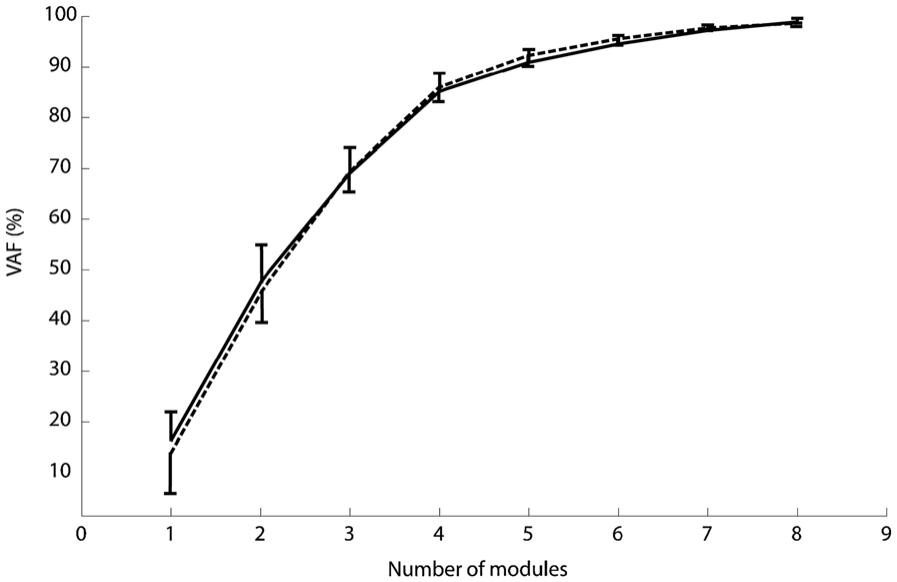
**Mean and standard deviation of the variation accounted for (VAF) with respect of the number of motor modules extracted. **No differences were observed for different combinations of speed/BWS in robot-aided walking. The dashed line represent the mean over all subjects and conditions for robot-aided walking, the solid line represents the overground walking trials of all subjects.

The average self-selected low speed while overground walking was 2.1 ± 0.6 km/h, which is approximately in the middle of the range of speeds tested during robot-aided walking (see in the following). The reconstruction quality of the muscular activation pattern with four modules, which was the chosen dimensionality according to the criteria described above, was on average 85.8±4.0% (Figure 2).

The motor modules extracted during overground walking were similar across subjects (mean similarity 0.67±0.07), although this similarity was lower than for Lokomat walking (see below for robot-aided walking).

**Figure 3:**
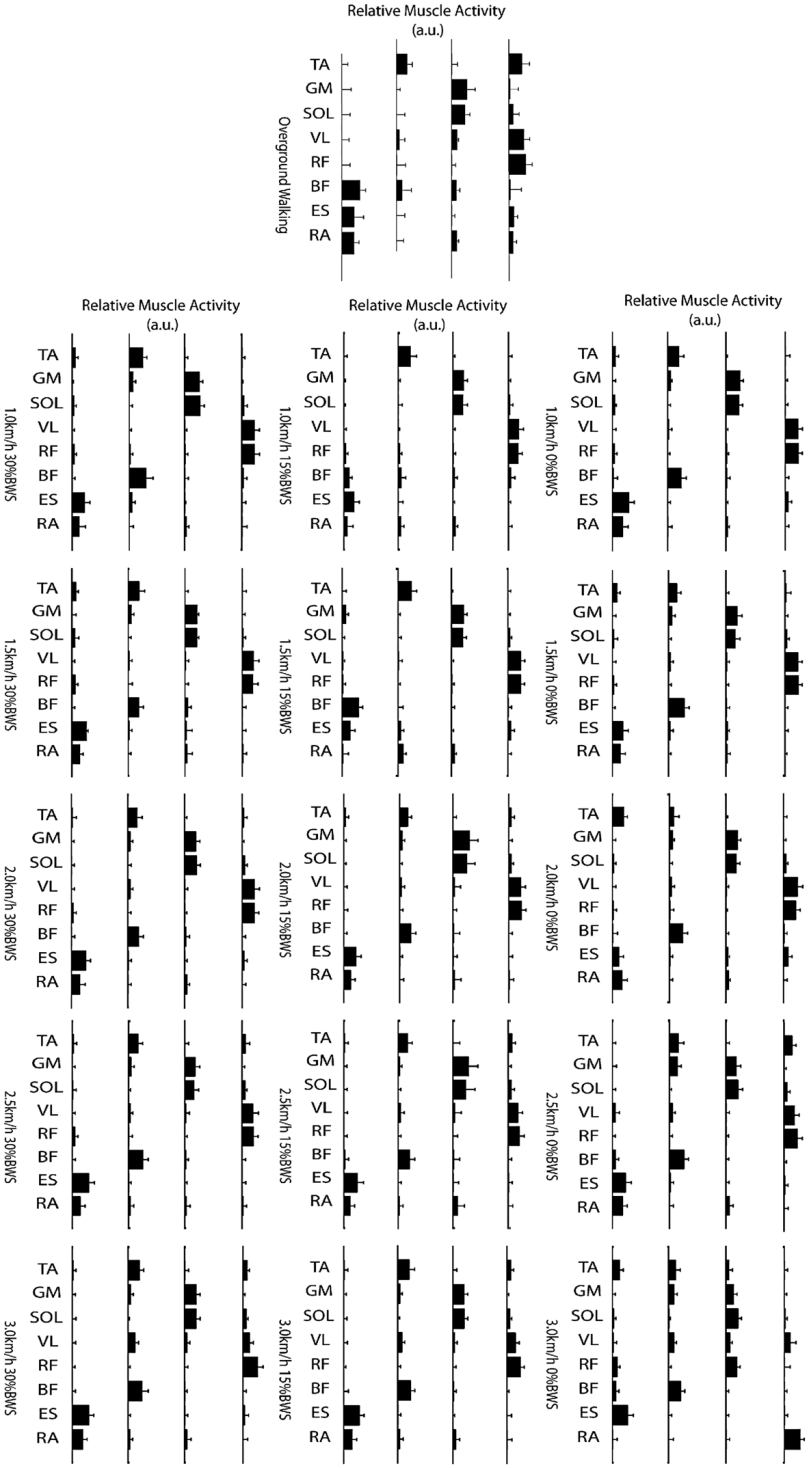
Motor modules for overground walking and robot-aided walking at different velocities and BWS (average and SD over all subjects).

**Figure 4:**
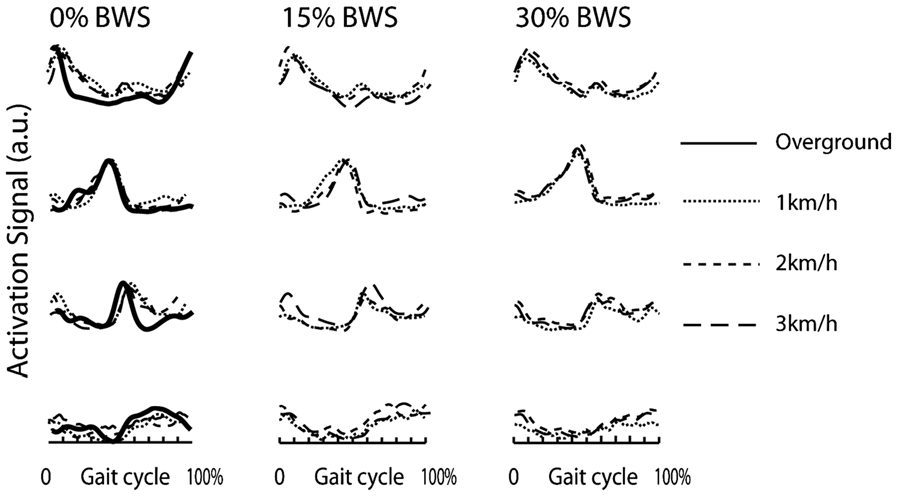
**Activation signals for different speeds and BWS for robot-aided walking and for overground walking (solid line), averaged over all subjects. **The activation signals for Lokomat walking were similar for different speeds and with respect to overground walking. The temporal displacement of peaks in activation signals is compatible with previously reported results from other studies [11-13,19].

Overground walking was characterized by simultaneous activation of TA, VL and RF, represented in the motor module 1, alternated to the activation of the GM and SOL on module 2. TA was also represented in module 3, whereas the BF muscle was mainly represented in the motor module 4 (Figure 3). The corresponding activation signals showed a burst-like activity (Figure 4), in agreement with previous results [12,13].

### Robot aided walking

**Figure 5:**
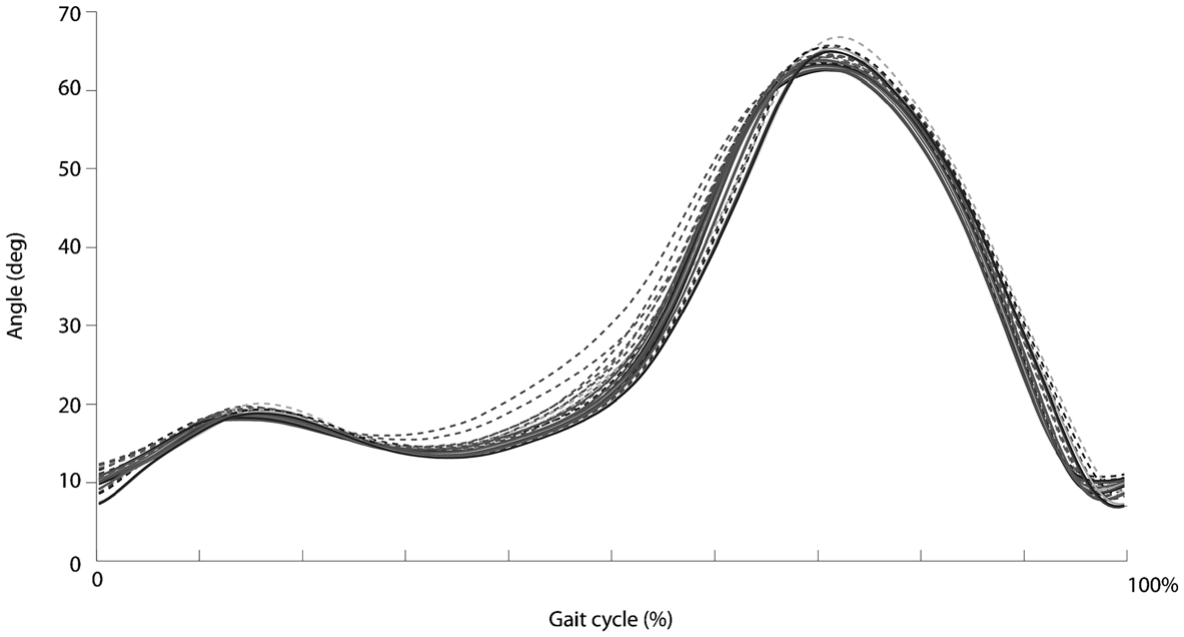
Angle profile during the gait cycle for all the combinations of speeds and BWS levels (mean values for each condition, solid lines; standard deviation of the mean for each condition, dashed line).

**Figure 6:**
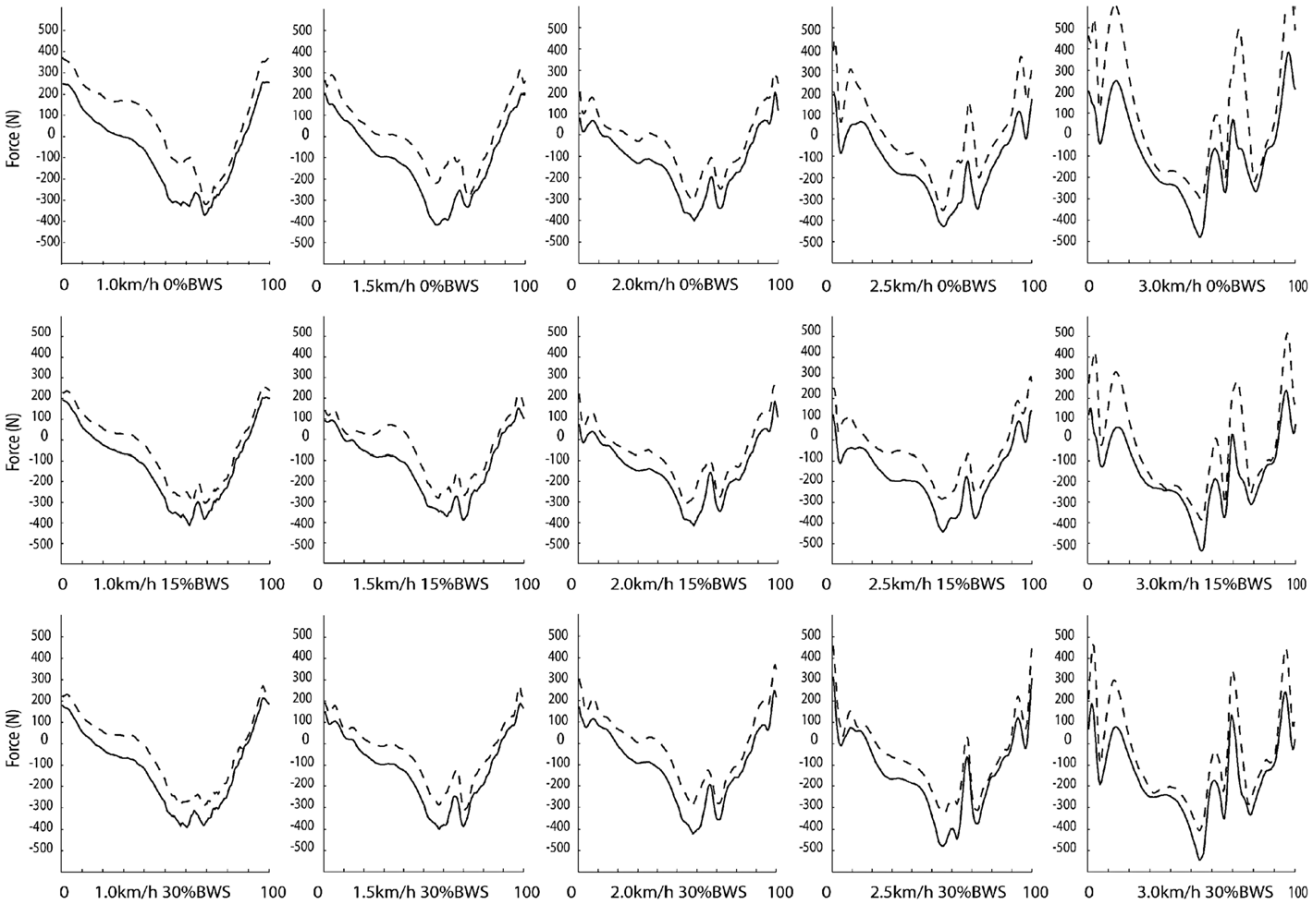
**Knee force profiles over different condition of speed and levels of BWS.** The solid traces represent the mean and the dotted lines the SD over all subjects.

Although the knee angle profile (normalized with respect to time, Figure 5) was not different among conditions the force profiles changed across conditions remarkably (see Figure 6). In particular, the 3.0 km/h speed resulted in a highly variable force profile at all levels of BWS: the average value of SD for force was ~95N among all conditions except for the speed 3km/h and ~136N for the conditions with speed at 3km/h.

#### Reconstruction quality

The reconstruction quality (VAF) for robot-aided walking depended on the number of modules and the dimensionality of control was 4, as obtained for the overground walking in this and previous studies [12,19]. The VAF was higher than 80% with 4 modules in all the conditions investigated (average 85.8±3.9%). Reconstruction quality with 4 modules was not different between overground and robot-aided walking. The trials at 3.0km/h for all the BWS levels resulted in a slightly greater, reconstruction quality with respect to the other conditions (89.0±3.3%).

#### Motor modules

The first motor module was characterized by the concomitant activation of knee extensors (VL, RF), the second by ankle plantar flexors (GM and SOL), and the third by the activation of plantar dorsiflexor (TA). The activity of BF was characterized by greater variability among conditions and was represented in module 3 and/or 4 (Figure 3).

During robot aided walking the motor modules in the 3km/h 0% BWS appeared different with respect to the other conditions: the first module was characterized by the activation of VL and RA, the second by a concomitant activation of ankle plantar flexors (GM, SOL) together with knee extensors (VL and RF), the third by a concomitant activation of TA, GM, VL and BF, and the fourth mainly by the activity of the TA, RF, BF and ES muscles (Figure 3). The similarity between motor modules extracted from overground and robot-aided walking was on average 0.70±0.09 (except for the 3km/h trials, where the mean similarity was 0.63±0.09, the difference was however not significant P = 0.68). An increase of BWS resulted in a more similar distribution of muscle weightings with respect of slower speeds: trials at 3.0km/h with 15% and 30% BWS, showed activation of knee extensors on module 1, plantar flexors on module 2, ankle dorsiflexor and knee flexor on module 3 and trunk activation on module 4.

#### Similarity of motor modules among subjects during robot-aided walking

Different subjects had similar motor modules in the same condition (mean similarity 0.76±0.03) for all conditions, except for the 3.0km/h 0% BWS, where the mean similarity was lower (0.64±0.32). Moreover, the motor modules for each subject were also similar across conditions (mean similarity 0.83±0.12).

The modules extracted from the complete dataset (all subjects) were very similar among conditions (mean similarity among conditions 0.93±0.04) except for the case 3.0km/h 0% BWS (average similarity with respect of the other conditions 0.64±0.1) (see Table 1).

**Table 1:** Motor modules similarity from the concatenation of all the subjects, with values lower or equal to 0.7 in bold


1.0km/h 0%BWS	-														
1.0km/h 15%BWS	0,91	-													
1.0km/h 30%BWS	0,99	0,90	-												
1.5km/h 0%BWS	0,98	0,85	0,99	-											
1.5km/h 15%BWS	0,78	0,92	0,81	0,86	-										
1.5km/h 30%BWS	0,99	0,91	0,99	0,98	0,75	-									
2.0lkm/h 0%BWS	0,91	0,79	0,91	0,95	0,94	0,90	-								
2.0km/h 15%BWS	0,99	0,89	0,98	0,99	0,78	0,98	0,90	-							
2.0km/h 30%BWS	0,99	0,91	0,99	0,98	0,76	0,98	0,89	0,99	-						
2.5km/h 0% BWS	0,94	0,84	0,94	0,94	0,75	0,92	0,86	0,96	0,95	-					
2.5km/h 15%BWS	0,97	0,91	0,97	0,96	0,75	0,96	0,86	0,99	0,99	0,96	-				
2.5km/h 30%BWS	0,98	0,89	0,98	0,98	0,77	0,98	0,89	0,99	0,99	0,96	0,99	-			
3.0km/h 0% BWS	**0,69**	**0,61**	**0,69**	**0,69**	**0,57**	**0,70**	**0,65**	**0,69**	**0,68**	**0,32**	**0,67**	0,71	-		
3.0km/h 15%BWS	0,97	0,90	0,96	0,96	0,73	0,96	0,86	0,98	0,98	0,96	0,99	0,99	**0,67**	-	
3.0km/h 30%BWS	0,96	0,87	0,95	0,95	0,73	0,94	0,86	0,97	0,97	0,96	0,98	0,97	**0,66**	0,99	-
	1.0km/h 0% BWS	1.0km/h 15% BWS	1.0km/h 30% BWS	1.5km/h 0% BWS	1.5km/h 15% BWS	1.5km/h 30% BWS	2.0lkm/h 0% BWS	2.0km/h 15% BWS	2.0km/h 30% BWS	2.5km/h 0% BWS	2.5km/h 15% BWS	2.5km/h 30% BWS	3.0km/h 0% BWS	3.0km/h 15% BWS	3.0km/h 30% BWS

The low values in similarity are all related to the 3.0km/h speed.

### Activation signals

**Figure 7:**
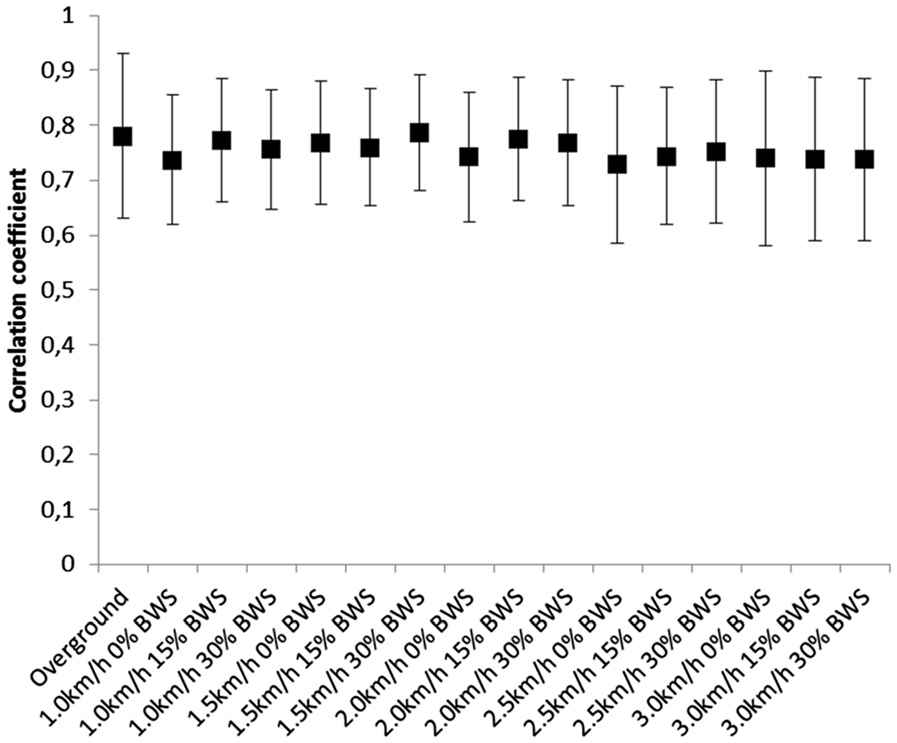
Average correlation of the activation signals among different individuals for each condition of robot-aided walking, compared with overground walking.

The activation signals during robot-aided walking showed the same burst-like structure as for the overground walking (Figures 3 and 4). The correlation of activation signals extracted from the overground walking trials of different subjects was 0.78 ± 0.15. This value was comparable with the correlation among activation signals of different subjects in different conditions (Figure 7) (average correlation among speed/BWS combinations 0.75±0.12).

The correlation between activation signals of overground walking and robot-aided walking for each subject and condition was on average 0.74±0.12, with no significant deviation for any combination of speed and BWS (P=0.70). This indicates a similarity between activation signals across type of walking, conditions and subjects.

## Discussion

Robot-aided walking could be described with a modular organization of muscular activation with the same dimensionality as overground walking. Moreover, the main characteristics of this organization were also similar in the two conditions. Specifically, an impulsive control of walking was evident in both conditions.

Four motor modules were sufficient to describe overground walking in agreement with results from previous studies on healthy subjects [12] and stroke patients [19,20]. In particular, the muscles investigated in this study were the same as in [20], where a dimensionality equal to 4 was found for overground walking. As generally accepted and reported by other authors [12,19], the dimensionality may slightly vary with the number of muscles investigated. A greater number of muscles, as in [11,13], may require a larger number of modules, although the general conclusion in all previous studies is for a limited dimensionality. Small differences in dimensionality found in different studies does not change the general physiological consideration that human locomotion can be effectively represented by a small set of basic components, robust with respect of inter-individual and inter-condition differences.

The similarity of motor modules and activation signals among different subjects for the same condition denotes a similar muscle activation paradigm among subjects. Moreover, the motor modules were similar when varying speed and BWS level, except for a relevant (but not statistically significant) difference for the trials at 3km/h. The peculiar composition of motor modules at 3km/h and 0% BWS is suggestive of a compensatory strategy of the trunk (with the alternate activation of RA and ES in module 1 and 4, respectively) and coactivation of ankle plantar and dorsiflexor muscles (modules 2 and 3), probably related to the difficulty of the subject to follow the movement of the machine at high speed –see below. The temporal behaviour of force traces (Figure 6) confirmed this interpretation. Although for overground walking a speed of 3.0km/h is in general suitable for healthy individuals, the motor modules identified in this study could be due either to the absence of hip rotational movement [53] and/or to a significant delay in the response of the orthosis (possibly due to both the inertial mass of the machine and more in general to the reaction time of the whole system) to the movement of the subject in impedance control mode [37]. The similarity of motor modules and activations signals in the other conditions, despite the already reported mechanical differences, could be addressed to the limitation of the investigated muscles to the sagittal plane only, where the movement of the machine is meant to be as similar as possible to natural walking.

The similarity of motor modules across different subjects and condition and with respect to overground walking evidences a common motor control strategy. This interpretation is in agreement with another study [54] where similar muscular activity profiles during walking on the Lokomat machine versus free walking were reported. However, another study [53] focusing on motor control during robot-aided walking with the Lokomat machine reported differences in muscular activation timing in the lower limbs during robot-aided walking. Those authors reported a difference in muscular activation of the TA, knee extensors and gluteus maximus with respect to treadmill walking. The disagreement with these results and a common modular organization observed in the present study may be due to technical (the absence of BWS harness and the level of guidance force which was not reported) and/or methodological differences (such as normalization procedure or electrode placement). Moreover an elastic bandage for dorsiflexion facilitation was used by Hidler et al. [53], which could influence the motor output, especially for the lower leg muscles.

For this experiment no explicit indication concerning the control of ankle joint was administered to the volunteers and, with the aim of mimicking the final stage of rehabilitation, no elastic bandage was applied to the ankle joint.

One of the main results of the present study is the evidence of a burst-like structure in activation signals, reported in this study for the first time in the case of robot-aided gait. This observation is in agreement with previous findings for treadmill [14,16,19,55] and overground walking at different velocities [13] and BWS levels [11], as well as in pathological condition [19,20]. It is hypothesized [11,13,55,56] that the action of the network in charge of distributing the muscular activity during locomotion strongly depends on proprioceptive feedback from limbs and vestibular system, and task constrains, whilst the timing network would be prominently regulated by locomotion cadence and speed. The afferent feedback contributes to adapt and modulate the activity of CPG to match the actual environment [57]. Although an important role in regulating the muscular output during locomotion is due to load changes [47,58,59], some authors reported that the effect of BWS is moderate when an adaptation (as in our case) occurred. According to this view, the presence of similar activation signals and motor modules despite changes in speed and BWS could be suggestive of a stereotyped activity of the distribution network integrating similar proprioceptive stimuli. In a previous study [11], a systematic phase shift of activation signals was observed and explained changes in the duration of the stance phase as a function of speed (ranging from 1 to 5 km/h in that case). Our results (Figure 4) did not show significant phase shifts of activation signals at varying speed, despite a small shift could be noticed for the activation signal 3. The reason for this apparent discrepancy can reasonably be addressed to the difference in range of velocities investigated in our study with respect to [11] Another study on locomotion modeling [47] reported differences in muscle weightings of synergistic muscles (i.e. soleus and gastrocnemius) at changing in BWS and body mass. The lack of differences in our results may be explained either by the small absolute value of BWS used for our experiments, and/or to the normalization process applied. However, we are more likely to address this phenomenon to the differences in experimental conditions since similar findings have been reported by another group [59] using the same normalization procedure, but higher levels of BWS. The study by McGowan [47], however, shows a minor effect of BWS and a rather more pronounced effect of increased weight and mass on EMG amplitude in those muscles. Moreover, the study reported that the temporal intervention of those muscles is robust at changing of loading condition.

The guidance force was set to 0% (free run mode) rather than a strict (position control with stiff joints) or partial guiding, for the purpose of directly testing the influence of the human-machine interface on motor control in the condition where the machine itself was behaving as transparent as possible. Although this is distant from the clinical practice (patients are often treated with up to 100% guidance force) the target of rehabilitation orthoses is to restore the ability to walk and the correct muscular activation pattern. By providing 0% guidance force, we analyzed the muscular activity when mimicking the ideal case of a fully recovered patient at the end of the rehabilitation process [39]. One prerequisite for the use of robotic training is that, in this ideal condition, walking in the robot corresponds to the physiological pattern of muscle activation as in free walking. If this condition is not met, the rehabilitation strategy by robot-aided walking would tend to generate locomotor patterns different from the normal walking. The results demonstrated that, although the number of degrees of freedom of the machine is less than in free walking, no differences in motor modules nor activation signals were reported for most of the conditions and muscles tested.

In conclusion, the results of this study indicate that robot-aided walking with the Lokomat has a modular organization with similar timing of the impulsive bursts of activity as overground walking. With respect to overground walking, however, the muscular activity during robot-aided gait was more stereotyped and similar among individuals, as concluded from the greater similarity of motor modules among individuals. This supports the view that robot-aided walking provides a therapeutic approach to restoring walking which is more repeatable and standardized than approaches based on exercising during overground walking. Although for a complete generalization more experiments with a wider range of BWS and guidance force would be desirable, the results pose the foundation for the use of robot-aided walking to restore the natural modular organization of walking.

## Abbreviations

BWS: Body weight support; EMG: Electromyographic; VAF: Variation accounted for; SSE: Sum of squared errors; SST: Total sum of squares; NMF: Non-negative matrix factorization; TA: Tibialis Anterior; GM: Gastrocnemius Medialis; SOL: Soleus; VL: Vastus Lateralis; RF: Rectus Femoris; BF: Biceps Femoris; RA: Rectus Abdominis; ES: Erector Spinae.

## Competing interests

The authors declare that they have no competing interests.

## Authors’ contribution

All authors have made substantial contributions to conception and design of the study, collection and interpretation of the data drafting and revising of the manuscript. All authors read and approved the final manuscript.
